# High-Fidelity Weak Signal Extraction for Coiled Tubing Acoustic Telemetry via Micro-Lever Suspension and Joint Denoising

**DOI:** 10.3390/s26082315

**Published:** 2026-04-09

**Authors:** Yingjian Xie, Hao Geng, Zhihao Wang, Haojie Xu, Hu Han, Dong Yang

**Affiliations:** 1 Hubei Key Laboratory of Oil and Gas Drilling and Production Engineering, Yangtze University, Wuhan 430100, China; 2024720459@yangtzeu.edu.cn (Y.X.); genghao@hilonggroup.com (H.G.); 2025720454@yangtzeu.edu.cn (Z.W.); jie07160705@163.com (H.X.); 2 School of Petroleum Engineering, Yangtze University, Wuhan 430100, China; 3 State Key Laboratory of Low Carbon Catalysis and Carbon Dioxide Utilization, Yangtze University, Wuhan 430100, China

**Keywords:** coiled tubing, acoustic telemetry, micro-lever, nonlinear dead zone, CEEMDAN, joint denoising

## Abstract

**Highlights:**

**What are the main findings?**

- A novel acoustic pickup probe based on the micro-lever principle with an arm ratio of 2.6 to 1 and a full pressure-balanced mechanism is developed to physically eliminate the contact dead zone of traditional probes and isolate low frequency common-mode interference via a lateral floating architecture.
- A CEEMDAN-wavelet threshold joint denoising model with a cross-correlation coefficient criterion is proposed, which adaptively screens intrinsic mode functions and achieves high-fidelity elimination of residual fluid turbulence noise in coiled tubing acoustic telemetry signals.

**What are the implications of the main findings?**

- The micro-lever suspension probe improves the radial breathing mode detection sensitivity by ~20.6 dB and effectively eliminates stick-slip friction noise during dynamic tripping, breaking through the physical limit of weak signal detection in deep well coiled tubing acoustic telemetry.
- The joint denoising algorithm increases the signal to noise ratio by an additional 16.9 dB under typical pumping conditions (0.5 bpm) with a normalized cross-correlation exceeding 0.96, providing a robust hardware-software integrated solution for high-fidelity weak signal extraction in downhole acoustic telemetry systems.

**Abstract:**

In Coiled Tubing (CT) acoustic telemetry, the reliability of surface signal reception is severely challenged by the “contact dead zone” of traditional probes and complex nonstationary environmental noise. To address these issues, this paper proposes a hardware-software integrated solution for high-fidelity signal extraction. In terms of hardware, a novel pickup probe based on the micro-lever principle is developed. By utilizing a pivoted lever structure with an optimized arm ratio of 2.6 to 1 and a full pressure-balanced mechanism, the design physically overcomes the contact dead zone inherent in traditional pressure-compensating probes and effectively isolates low frequency common-mode interference through a lateral floating architecture. In terms of software, a joint denoising model combining Complete Ensemble Empirical Mode Decomposition with Adaptive Noise and wavelet thresholding is proposed. A cross-correlation coefficient criterion is introduced to adaptively screen intrinsic mode functions and eliminate residual fluid turbulence noise. Field experiments on a 1500 ft full-scale circulation loop demonstrate that the proposed probe improves the detection sensitivity of the radial breathing mode by approximately 20.6 dB compared to the baseline, while effectively eliminating stick-slip friction noise during dynamic tripping. Furthermore, the joint algorithm increases the Signal to noise Ratio by an additional 16.9 dB under typical pumping conditions of 0.5 bpm, with a normalized cross-correlation exceeding 0.96. These results verify that the proposed method effectively solves the bottleneck of weak signal detection in deep wells, providing robust technical support for CT telemetry operations.

## 1. Introduction

With the global exploration and development of oil and gas resources moving toward deep, ultra-deep wells and long horizontal shale gas wells, the downhole operating environment has become increasingly complex, imposing higher requirements on real-time data transmission technologies for measurement while drilling (MWD) [1]. In response to this trend, Su et al. [2] pointed out that the innovation of measurement and control while drilling is the key to addressing complex geological and engineering challenges. Coiled tubing (CT) technology has achieved remarkable progress in unconventional oil and gas drilling due to its advantages such as operation under pressure, fast tripping speed, and large inner diameter [3]. A comparison of shale oil and gas exploitation technologies between China and the United States shows that this technology has become a core method for stimulation and workover operations [4]. In addition, combined with distributed acoustic sensing technology, coiled tubing has demonstrated great application potential in vertical seismic profile logging [5]. However, conventional memory logging cannot meet the demands of real-time decision-making; wired cables face risks of difficulty in pushing and easy winding in long horizontal sections, and mud pulse telemetry suffers from low transmission rates under aerated drilling fluid or two-phase flow conditions [6]. Meanwhile, Pang et al. [7] further confirmed through their research on modulation optimization that mud pulse signals are highly prone to waveform distortion and instability in complex media. In contrast, studies in downhole control engineering indicate that acoustic telemetry technology uses the pipe string itself as an elastic wave channel, requiring no pre-installed cables and being unaffected by drilling fluid media [8]. From the perspective of rod and pipe string mechanics, this technology can theoretically realize full-bore and high-bandwidth data transmission, and is regarded as a key technology to solve the problem of monitoring while drilling under complex working conditions in deep wells [9]. Despite its broad prospects, acoustic signals transmitted in liquid-filled pipe strings of several thousand meters suffer from severe energy attenuation and channel distortion [10]. The signals reaching the surface are often as weak as micro-gravity levels and are easily submerged in strong background noise generated by high-pressure pumping and mechanical driving at the wellhead [11]. Therefore, achieving high-fidelity extraction of weak acoustic signals in a low signal to noise ratio (SNR) environment has become the core bottleneck restricting the large-scale application of this technology.

A large number of studies have been conducted by scholars worldwide on the attenuation mechanism and channel characteristics of acoustic signals. Ma et al. [12] established an acoustic transmission model for coiled tubing with couplings based on one-dimensional wave theory, pointing out that the periodic coupling structure produces a comb-filter effect, resulting in passband and stopband characteristics for signals in specific frequency bands. Zhao et al. [13] further considered the coupled dissipation mechanism of fluid viscosity and pipe wall friction and quantified the exponential attenuation law of acoustic waves in deep wells. Their experimental data show that the acoustic amplitude attenuation can exceed 60 dB at a well depth of 3000 m. In view of this physical attenuation characteristic, current solutions mainly focus on two aspects: digital signal processing at the software level and sensor optimization at the hardware level.

To mitigate the aforementioned physical signal attenuation, various time-frequency analysis algorithms in digital signal processing have been introduced to separate weak signals from strong noise backgrounds. Traditional infinite impulse response and finite impulse response filters are computationally simple but perform poorly in dealing with nonstationary fluid turbulence noise [14]. To this end, Torres et al. [15] proposed an adaptive threshold denoising method based on wavelet packet transform to effectively suppress drill string vibration noise. Nevertheless, its denoising performance highly depends on the prior selection of wavelet basis functions and decomposition levels, resulting in limited adaptability.

To overcome the limitations of fixed basis functions, adaptive decomposition algorithms represented by empirical mode decomposition have attracted considerable attention in recent years. For instance, Colominas et al. [16] applied ensemble empirical mode decomposition to process acoustic signals while drilling, solving the mode mixing problem to a certain extent, but the residual white noise reduced the fidelity of the reconstructed signal. In response, Sahli et al. [17] proposed the complete ensemble empirical mode decomposition with adaptive noise (CEEMDAN) algorithm, which achieved higher decomposition accuracy and completeness by adding adaptive noise and calculating a unique residual. However, Pagtalunan et al. [18] pointed out in applying variational mode decomposition for acoustic denoising that relying solely on algorithms can hardly handle strong mechanical co-channel interference overlapping with the signal frequency band, and when the input SNR is below a certain threshold, it is difficult for any algorithm to recover effective information from heavy noise. This indicates that the optimization of back-end algorithms alone has reached its limit, and there is an urgent need to improve the pickup quality of front-end physical signals.

In hardware pickup and sensor technology, improving detection sensitivity is the research focus. At present, piezoelectric ceramic accelerometers or hydrophones are widely used as sensitive components in industry. Zha et al. [19] developed a high-temperature resistant piezoelectric acoustic receiving transducer, improving charge sensitivity by optimizing the piezoelectric stack structure. Xu et al. [20] designed a wellhead acoustic detection system based on fiber Bragg grating, achieving satisfactory test results in the laboratory environment using the high sensitivity of optical fibers. However, existing studies mostly focus on the materials and circuit gains of the sensors themselves, ignoring the key link of the mechanical coupling mechanism between the probe and the pipe string under test. In actual coiled tubing operations, to balance wellhead high pressure, traditional probes generally adopt a hydraulic piston-type pressure compensation structure, in which rollers are pressed against the pipe wall with large pre-tightening force [21]. According to contact mechanics theory, excessive normal force leads to a sharp increase in static friction at the contact interface, forming a significant dead zone in displacement response [22]. Dynamic analysis by Zhao et al. [23] shows that when the weak vibration displacement of the pipe wall is lower than the dead zone threshold, the sensor severe loses responsiveness. Such physical signal truncation cannot be compensated by any back-end amplifier circuit or algorithm.

In summary, although great progress has been made in acoustic channel modeling and digital denoising algorithms, there is still a lack of systematic solutions to the signal pickup failure caused by mechanical dead zones due to high contact stress and common-mode interference due to rigid connections in the high-pressure operating environment of coiled tubing. Existing direct-pressure probes are difficult to break through the detection limit of weak signals at the physical source, becoming a shortcoming restricting the practical application of acoustic telemetry in deep wells.

To address these interconnected challenges, this research establishes a multi-level synergistic architecture that creates a rigorous logical progression from physical source activation to adaptive numerical cleaning. The core innovation lies in the systematic integration of hardware pre-amplification and software fine denoising. At the physical layer, a micro lever suspension probe is designed to break through the contact dead zone by converting weak radial vibrations into amplified axial signals, effectively activating the sensor response before signal loss occurs. At the acquisition layer, a high dynamic range signal chain is implemented to quantize the micro gravity features without saturation under high pressure interference. Finally, at the algorithmic layer, a joint CEEMDAN wavelet model performs secondary purification of nonstationary noise that remains after mechanical isolation. This cascaded logic ensures that the hardware gain provides a high-quality analog baseband while the software gain guarantees robust digital demodulation, thereby providing a comprehensive solution for weak signal extraction in deep well coiled tubing telemetry.

## 2. Materials and Methods

### 2.1. Mechanism and Signal Model of Acoustic Channel in Coiled Tubing

#### 2.1.1. Acoustic Attenuation Mechanism and Surface Reception Dead Zone

The acoustic wave propagation in coiled tubing constitutes a fluid structure coupled elastic transmission process. This analytical framework is specifically parameterized for the coiled tubing environment by integrating the fluid and structure interaction of slurry under high pressure within a continuous elastic channel. Unlike generic acoustic models, the integrated attenuation coefficient incorporates the dissipation mechanisms depending on frequency that are unique to the thin wall geometry of coiled tubing. This serves as a critical basis for identifying the signal carrier frequency in deep well telemetry. Field data demonstrate that due to this coupled dissipation and the impedance mismatch at tubing couplings, the radial vibration acceleration of the pipe wall at the wellhead typically attenuates to the micro gravity level. This extreme physical attenuation imposes stringent sensitivity requirements on surface detection equipment, necessitating mechanical pre-amplification prior to digital quantization.

The physical essence of surface signal detection lies in picking up the weak radial displacement of the pipe wall. However, the conventional hydraulic piston-type probe introduces an obvious Coulomb friction dead zone due to over-compensation [24].

Let the actual radial vibration displacement at the pipe wall be x(t), and the displacement picked up by the probe accelerometer be y(t). A dynamic model of the contact interface between the probe and the pipe wall is established. When the preload $F_{\mathrm{pre}}$ at the contact interface is excessively large, the system exhibits typical dead-zone nonlinearity [25]. Its input-output relationship can be mathematically modeled as:

(1) $$y\left(t\right)=\left\{\begin{array}{ll} x\left(t\right)-\delta , & x\left(t\right)>\delta \\ 0, & \left|x\left(t\right)\right|\leq \delta \\ x\left(t\right)+\delta , & x\left(t\right)<-\delta \end{array}\right.$$

where δ is the dead-zone threshold determined by static friction, satisfying:

(2) $$\delta =\frac{\mu_{s}F_{\mathrm{pre}}}{k}$$

where $\mu_{s}$ is the static friction coefficient; $F_{\mathrm{pre}}$ is the contact preload, Pa; k is the contact stiffness.

It can be seen from the equation that when the pipe wall vibration |x(t)| excited by weak acoustic waves is smaller than the friction dead-zone threshold δ, the probe output y(t)=0. This mathematically explains why conventional probes completely fail under weak signals. This is also the theoretical basis for the lateral suspension and micro-lever structure proposed in this paper, that is, reducing $F_{\mathrm{pre}}$ and amplifying x(t) through lever gain via mechanical structure design, so as to overcome the dead-zone threshold δ.

#### 2.1.2. Dynamic Modeling and Frequency Response Analysis of the Micro-Lever Structure

To overcome the contact dead-zone threshold, this paper proposes a mechanical amplification mechanism based on the micro-lever principle. This mechanism takes the coiled tubing as the acting object and converts the weak radial breathing vibration of the pipe wall into axial compression motion of the piezoelectric ceramic stack.

- Geometric gain design based on moment balance

Let the fulcrum of the lever be O, the arm length $L_{1}$ from the pipe wall contact point R to the fulcrum, and the arm length from the center A of the piezoelectric stack to the fulcrum be $L_{2}$.

Considering only the moment balance under quasi-static conditions, when the pipe wall produces a radial displacement input $x_{in}$, the theoretical output displacement $x_{out}$ acting on the piezoelectric element can be expressed as:

(3) $$x_{\mathrm{out}}=\frac{L_{2}}{L_{1}}\cdot x_{\mathrm{in}}$$

The basic geometric gain coefficient $G_{\mathrm{geo}}$ is defined as:

(4) $$G_{\mathrm{geo}}=20\log_{10}\left(\frac{L_{2}}{L_{1}}\right)$$

In this design, the arm ratio $L_{2}/L_{1}$ is increased from 1.0 in conventional designs to 2.6 by optimizing the probe geometry. Theoretical calculations show that the structure can provide a basic geometric gain of approximately 8.3 dB. This amplifies weak signals originally falling into the dead zone, thereby activating the response capability of the sensor at the physical level.

- Frequency response dynamics correction considering inertia and stiffness

However, under acoustic excitation at relatively high frequencies such as 960 Hz, the rotational inertia of the probe arm and the stiffness of the piezoelectric stack cannot be neglected, and the simple static model requires dynamic correction. The proposed dynamic model departs from conventional leverage functions by addressing the specific mechanical impedance matching between the flexible pipe wall and the high-stiffness piezoelectric stack. It explicitly accounts for the boundary conditions imposed by the radial breathing mode, where the force transmission is modulated by the contact stiffness of the lever pivot, a distinctive feature required to extract micro-gravity signals that would otherwise be masked by high-pressure wellhead friction. Consequently, a single degree of freedom rotational dynamic model is established. By introducing the equivalent rotational inertia of the system and the equivalent stiffness of the piezoelectric element, the dynamic amplitude frequency response function is derived:

(5) $$\left|H\left(\omega \right)\right|=\frac{x_{\mathrm{out}}}{x_{\mathrm{in}}}=\frac{L_{2}}{L_{1}}\cdot \frac{\beta }{\sqrt{{\left(1-\lambda^{2}\right)}^{2}+{\left(2\zeta \lambda \right)}^{2}}}\frac{1}{2}$$

where β=0.92 is the stiffness coupling coefficient, reflecting the weak cancellation of the lever gain by the piezoelectric stack reaction force; λ is the ratio of the operating frequency *ω* to the natural frequency $\omega_{n}$; ζ is the damping ratio.

Substituting the physical parameters of the probe, the natural frequency $\omega_{n}$ of the system is calculated to be 2850 Hz. At the carrier frequency of 960 Hz, λ is 0.34. Although real mechanical systems inherently possess multiple natural frequencies, structural analysis shows the higher order modal frequencies of this micro lever exceed 10 kHz. Since the 960 Hz acoustic carrier frequency is profoundly lower than the 2850 Hz fundamental frequency, the dynamic response is entirely dominated by the first order mode. Therefore, the dynamic contributions of unexcited higher order modes can be safely neglected, validating the single degree of freedom simplification in Equation 5. The analysis shows that although the stiffness coupling results in β<1, the denominator effect of the inertia term provides dynamic compensation because the system operates in the sub-resonant region. The corrected theoretical dynamic gain is approximately 9.5 dB. This result not only verifies that the system is far from the resonance point to ensure the linearity of the gain but also explains the high-efficiency amplification characteristic of the micro-lever structure in the acoustic frequency band from the perspective of dynamics.

#### 2.1.3. Dynamic Vibration Isolation Characteristics of the Lateral Suspension System

In addition to signal amplification, suppressing low frequency rigid vibration transmitted from the wellhead blowout preventer (BOP) is another key to improving the signal to noise ratio. Conventional probes are rigidly connected to the BOP, resulting in direct through transmission of common-mode interference. The lateral suspension structure designed in this paper is dynamically equivalent to a single-degree-of-freedom mass-spring-damping system.

Let the mass of the suspension system be *m* and the equivalent stiffness be $k_{s}$. The displacement transmissibility $T_{r}$ of the system to base vibration is given by:

(6) $$T_{r}\left(\omega \right)=\sqrt{\frac{1+{\left(2\zeta r\right)}^{2}}{{\left(1-r^{2}\right)}^{2}+{\left(2\zeta r\right)}^{2}}}$$

where $r=\omega /\omega_{n}$ is the frequency ratio, $\omega_{n}=\sqrt{k_{s}/m}$ is the natural frequency of the system, Hz; ζ is the damping ratio.

By adjusting the stiffness $k_{s}$ of the suspension spring, the natural frequency $\omega_{n}$ of the system is designed to be below the acoustic communication frequency band. When the external interference frequency is much higher than $\omega_{n}$, the probe moves together with the BOP. For high frequency acoustic signals much lower than $\omega_{n}$, the displacement transmissibility $T_{r}$ approaches zero, thus achieving effective mechanical isolation from the rigid background noise of the BOP.

### 2.2. Development of a Ground Detection Device for Weak Acoustic Signals

To address the issues of contact dead zone and common-mode interference, this paper develops a laterally suspended acoustic detection device based on the micro-lever principle. The device mainly consists of three components: a lateral suspension vibration isolation mechanism, a micro-lever gain unit, and a full pressure-balanced contact module.

#### 2.2.1. Structure and Limitations of Pressure-Compensated Probe

Current coiled tubing acoustic telemetry systems generally adopt traditional pressure-compensated pickup probes. To clarify the necessity of the novel probe design, this section combines the physical structure of the probe and the contact dynamics model to deeply analyze its mechanism defects in weak signal detection.

- Rigid direct connection structure and unit mechanical gain limitation

**Figure 1:**
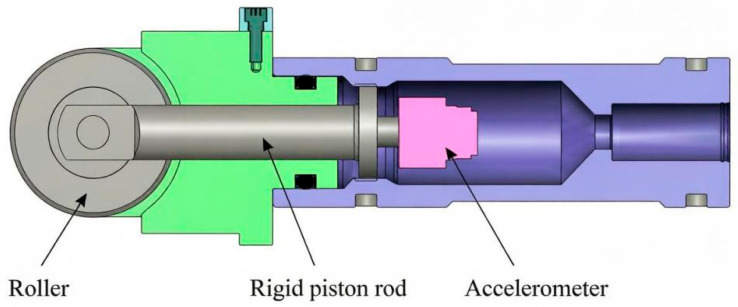
Structure of pressure-compensated pickup probe.

Traditional pressure-compensated probes usually adopt a single-degree-of-freedom linear piston configuration. As shown in Figure 1, the core components of the probe mainly include a front coupling roller, a middle rigid piston rod, and a rear accelerometer.

As shown in the figure, the roller, as the coupling interface in direct contact with the coiled tubing surface, is responsible for intercepting and picking up the radial vibration of the pipe wall. The rigid piston rod acts as a transmission medium and performs translational movement in the radial direction. The accelerometer is directly mounted at the rear of the piston rod to sense the incoming vibration energy.

From a kinematic perspective, the roller, piston rod, and accelerometer are tightly connected and move synchronously through a rigid structure. Neglecting the elastic deformation of the material, the transmission relationship satisfies:

(7) $$x_{\mathrm{sensor}}(t)=x_{\mathrm{in}}(t)$$

where $x_{in}(t)$ is the radial displacement input from the pipe wall, mm; $x_{sensor}(t)$ is the response displacement at the sensor, mm. The mechanical gain coefficient ***ε*** of the probe is defined as the ratio of output displacement to input displacement, which is given by:

(8) $$\varepsilon =\frac{x_{\mathrm{sensor}}}{x_{\mathrm{in}}}\approx 1$$

It can be seen that this type of probe is essentially a direct coupling structure with a constant mechanical gain coefficient of 1. This means the structure provides no physical amplification for weak breathing mode signals. The final detection sensitivity of the system is completely limited by the charge sensitivity of the rear-end piezoelectric ceramic, and the original signal to noise ratio cannot be improved via mechanical structure optimization.

- Friction noise and nonlinear dead zone under pressure coupling

**Figure 2:**
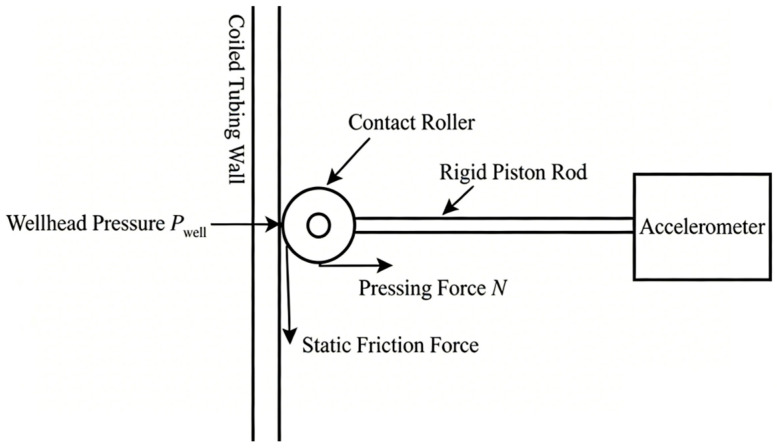
Schematic diagram of the single-degree-of-freedom dynamic model of the pressure-compensated pickup probe.

In addition to the lack of mechanical gain, the passive fluid pressure compensation mechanism adopted in conventional probes exhibits significant dynamic drawbacks under high-pressure working conditions. As shown in Figure 2, the probe can be dynamically regarded as a single-degree-of-freedom linear translation system subjected to multiple force fields.

(1) Background Noise Deterioration Caused by Excessive Contact Pressure Coupling

The probe shown in Figure 2 uses wellbore fluid pressure to drive the piston. Its original design intention is to use the well pressure to generate a reaction force to overcome the ejection effect under high-pressure environments. However, this passive compensation design results in a linear positive correlation between the pressing force of the roller on the pipe wall and the wellhead pressure:

(9) $$N=P_{\mathrm{well}}\cdot A_{\mathrm{piston}}+F_{\mathrm{pre}}$$

where N is the contact force between the roller and the pipe wall, Pa; $P_{\mathrm{well}}$ is the wellhead pressure, Pa; $A_{\mathrm{piston}}$ is the pressure-bearing area of the piston, cm^2^; $F_{\mathrm{pre}}$ is the initial preload, Pa. It can be seen from the above equation that as the wellhead operating pressure increases, the contact force applied to the pipe wall increases linearly. According to Coulomb’s friction law, this over-compensation effect causes a manifold increase in random friction noise at the contact interface, which masks weak effective acoustic signals and results in a sharp degradation of the signal to noise ratio.

(2) Nonlinear Response Dead Zone Induced by Static Friction

In weak signal detection, the large normal force on the contact surface not only introduces noise but also causes a severe nonlinear dead zone. As described in the nonlinear dead zone model established in Section 2.1.2, the displacement dead zone threshold determined by static friction is proportional to the preload on the contact surface.

In deep well and high-pressure conditions, the contact interface remains under extremely high preload for a long time due to the pressure-coupling mechanism, which significantly increases the dead zone threshold δ. When the amplitude of the weak acoustic vibration reaching the surface is smaller than this elevated threshold, the sensor falls into the stick regime. As a result, the probe and the pipe wall become relatively stationary, and the response output is physically truncated to zero.

#### 2.2.2. Mechanical Structure Design of the Lateral Suspension Micro-Lever Probe

To address the problems of contact dead zone and common-mode interference coupling in conventional probes, a novel lateral suspension pickup probe is developed in this paper. Different from the traditional piston structure, the proposed probe is designed based on a pivoted lever configuration. Through the optimization of dynamic structure, the mechanical pre-amplification of weak signals and physical isolation of background noise are realized.

- Overall architecture and working principle

**Figure 3:**
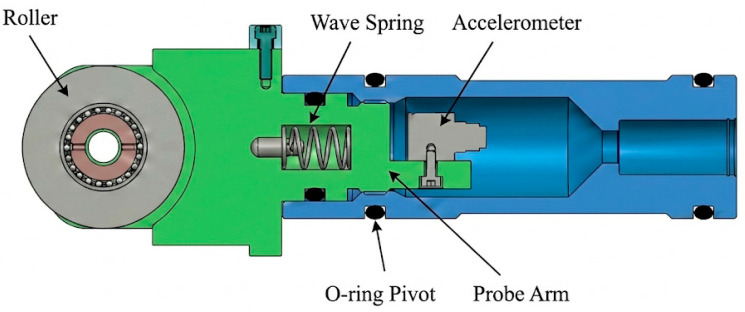
Lateral suspended micro-lever pickup probe structure.

The mechanical structure of the new probe is shown in Figure 3. The components abandon the rigid piston rod and adopt a lever-type topology connected by a precision pivot. The core components mainly include front coupling roller, probe arm, O-ring pivot, wave spring, rear accelerometer, and outer floating housing.

The working mechanism of the probe is based on the floating lever effect under pressure balance. In operating condition, the whole device is in a pressure-balanced suspended state. The internal wave spring provides a constant preload force, which drives the probe arm to rotate slightly around the O-ring pivot, thus pressing the front roller against the coiled tubing surface with a stable contact force. When the downhole acoustic signal propagates to the surface, the weak radial breathing vibration of the pipe wall actuates the roller, which further drives the probe arm to swing around the pivot. This small angular displacement is transmitted and amplified by the lever arm and is finally picked up as a high signal to noise ratio tangential acceleration signal by the accelerometer mounted at the end of the long arm.

- Design of the lateral suspension vibration isolation mechanism

**Figure 4:**
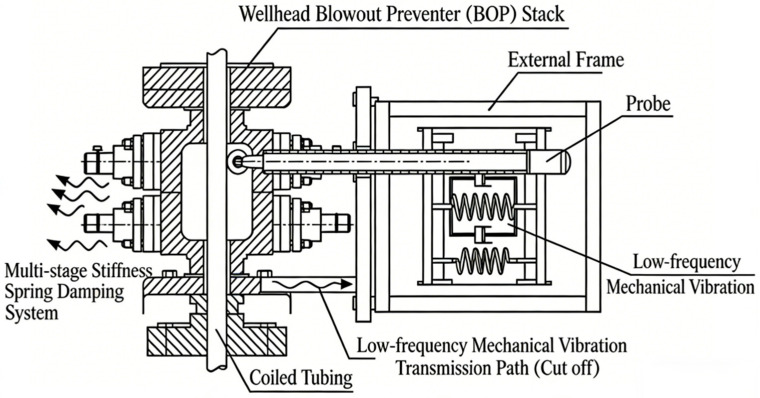
Lateral suspension pickup probe mounting structure.

To cut off the transmission path of strong low frequency mechanical vibration from the wellhead blowout preventer stack to the sensor, a lateral suspension mounting structure is proposed in this design, as shown in Figure 4. The probe body is no longer rigidly fixed by flanges but is suspended on the outer frame of the BOP via a multi-stage stiffness spring-damper system.

The suspension system dynamically forms a mechanical high-pass filter with dual frequency-response characteristics:

(1) Low frequency following characteristic (<5 Hz): For low frequency, large-amplitude vibrations caused by string movement or pump strokes, the suspension mechanism drives the probe to move in a following manner, maintaining a constant relative position between the probe and the string. This effectively avoids impact noise generated by rigid confrontation.

(2) High frequency vibration isolation characteristic (>500 Hz): For the effective signal band of acoustic telemetry, the suspension spring behaves as a soft connection, which greatly attenuates the background common-mode interference transmitted from the BOP body. In this way, the signal to noise ratio is initially improved from the physical transmission path.

- Parameter optimization of the micro-lever gain unit

**Figure 5:**
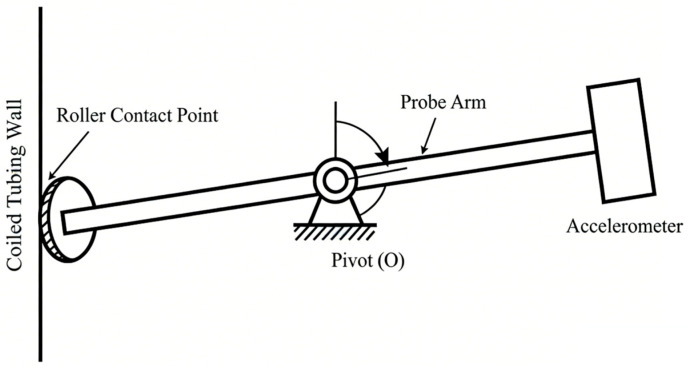
Schematic diagram of asymmetric lever transmission mechanism.

As a core component of the device that overcomes the contact dead zone and achieves physical amplification of weak signals, the geometric parameter tuning of the micro-leverage amplification unit directly determines the front-end pickup sensitivity of the system. As shown in Figure 5, this study designs an asymmetric lever transmission mechanism rotating around a precision bearing pivot. It aims to compensate for the insufficient charge sensitivity of conventional piezoelectric sensors under weak vibrations using the geometric amplification of the mechanical structure. In the selection of key geometric parameters, considering both the radial vibration impedance of the coiled tubing outer wall and the installation space constraints of the ground device, the short input arm $L_{1}$ is set to 19.05 mm to ensure high frequency radial stiffness at the contact interface. Meanwhile, to maximize the displacement amplification ratio, the long output arm $L_{2}$ is determined as 49.53 mm.

**Figure 6:**
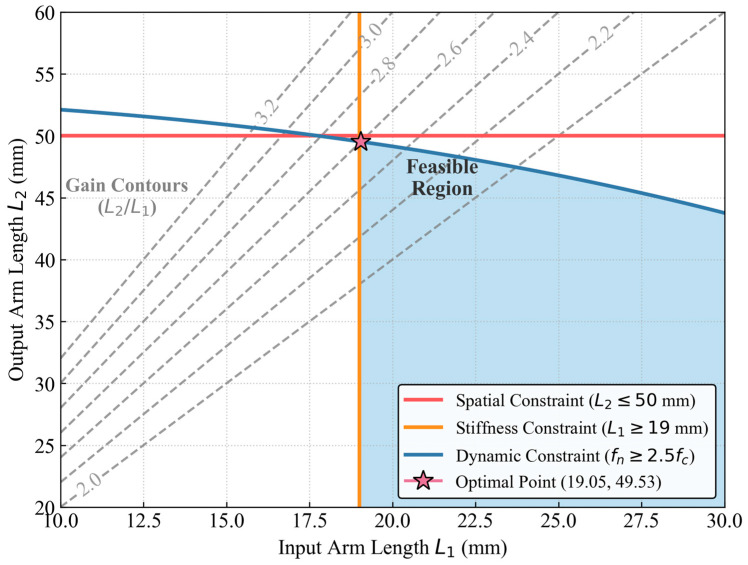
Design space optimization contour illustrating the feasible parameter region bounded by mechanical constraints and the convergence point for the lever arms.

To determine the precise dimensions of the mechanism shown in Figure 5, its geometric design is formulated as a multi-objective constrained optimization problem rather than an empirical selection. The primary mathematical objective is to maximize the geometric displacement gain which equals $L_{2}$ divided by $L_{1}$. This maximization is subjected to three strict physical boundary conditions. First, the spatial constraint of the wellhead blowout preventer frame dictates that the output arm length must satisfy $L_{2}$ ≤ 50 mm. Second, to prevent bending mode interference at the contact interface, the input arm must exceed a minimum structural stiffness threshold of $L_{1}$ ≥ 19 mm. Third, the dynamic rotational inertia constraint requires the system natural frequency to be at least 2.5 times the acoustic carrier frequency of 960 Hz to ensure operation within the flat sub resonant region. To accurately solve this optimization problem, a numerical iteration algorithm was employed to map the design space and evaluate the constraint intersections as illustrated in Figure 6. The numerical solution ultimately converged at the optimal parameter set of $L_{1}$ = 19.05 mm and $L_{2}$ = 49.53 mm, precisely yielding the theoretical 2.6 to 1 geometric ratio.

Through an in-depth mechanical mechanism analysis, this asymmetric lever structure not only provides a basic geometric gain of approximately 8.3 dB via a 2.6:1 force-arm ratio, but more importantly, it changes the driving threshold for signal pickup at the physical source. According to the nonlinear dead-zone model, the significant mechanical amplification in this design greatly reduces the minimum driving displacement required at the input to overcome static friction compared with traditional direct-contact probes, thus effectively activating the response capability of the sensor. Combined with dynamic correction analysis, within the acoustic carrier frequency band of 960 Hz, the system operates in the sub-resonant region, and the denominator effect of the inertial term further compensates the dynamic gain up to 9.5 dB. This physical hard-gain pre-amplification scheme effectively solves the problem of signal truncation caused by weak micro-G vibrations easily falling into the friction dead zone. While improving the detection limit of the system, it also significantly reduces the dependence on high-gain amplification characteristics of the back-end circuits, laying a solid hardware foundation for subsequent high-fidelity signal extraction under complex background noise.

- Full pressure balance and constant contact force control

To solve the problem of probe “overpressure locking” that easily occurs under high-pressure conditions in deep wells, full pressure balance technology is adopted in this design. A pressure introduction channel is arranged inside the structure to introduce the wellbore fluid pressure $P_{\mathrm{well}}$ into the back-pressure chamber of the probe piston in real time.

Assuming the pressure-bearing area of the piston is S, the thrust generated by the fluid is:

(10) $$F_{\mathrm{fluid}}=P_{\mathrm{well}}\cdot S$$

where $F_{\mathrm{fluid}}$ is the thrust generated by the fluid, Pa; $P_{\mathrm{well}}$ is the wellhead pressure, Pa; S is the pressure-bearing area of the piston, cm^2^. This thrust is fully counterbalanced by the internal equilibrium pressure. At this point, the contact force $F_{\mathrm{contact}}$ between the probe roller and the pipe wall no longer varies with wellbore pressure, but is independently controlled only by the rear pressure-regulating spring:

(11) $$F_{\mathrm{contact}}=k_{\mathrm{spring}}\cdot \Delta x_{\mathrm{spring}}$$

where $F_{\mathrm{contact}}$ is the contact force, Pa; $k_{\mathrm{spring}}$ is the stiffness coefficient of the pressure-regulating spring; $\Delta x_{\mathrm{spring}}$ is the compression displacement of the spring, cm.

Through this decoupling design, even if the wellhead pressure fluctuates drastically within the range of 0–68.95 MPa, the normal contact force is always maintained in the preset optimal linear region. This not only avoids the friction dead zone caused by excessive pressure in conventional probes, but also prevents signal decoupling due to insufficient pressure.

#### 2.2.3. High Dynamic Range Analog Front-End and Data Acquisition System

Following the mechanical amplification by the micro lever unit, the piezoelectric element converts the physical vibration into a corresponding electrical charge. The design of the subsequent high dynamic range analog front end and data acquisition system is fundamentally driven by the extreme amplitude disparity between the intense pump interference and the attenuated acoustic carrier.

To quantify this hardware requirement, a dynamic range calculation model is established. The required dynamic range DR for the acquisition system is formulated as:

(12) $$DR=20\log_{10}\frac{A_{\max}}{A_{\min}}+R_{\mathrm{margin}}$$

where $A_{\max}$ represents the peak amplitude of the low frequency pump noise, $A_{\min}$ denotes the threshold amplitude of the target acoustic signal, and $R_{\mathrm{margin}}$ is the required engineering redundancy margin. Field measurements indicate that the noise to signal amplitude ratio often exceeds a factor of one hundred thousand in deep well environments. According to the established model, this necessitates a minimum system dynamic range exceeding 100 decibels. Consequently, traditional 16-bit acquisition systems are prone to severe signal clipping or quantization noise masking.

To overcome this fundamental bottleneck, this section constructs a complete signal conditioning chain. This architecture integrates a high impedance charge amplifier with a programmable gain network and a 24-bit delta sigma analog to digital converter, providing a theoretical dynamic range of 144 decibels. It ensures that the micro gravity acoustic features are linearly quantized without saturating the signal chain under severe mechanical vibrations. The specific implementation of this architecture is detailed below.

- Isolated coupling model and wideband charge amplification mechanism

Piezoelectric ceramic sensors are essentially high-impedance, capacitive signal sources, whose equivalent circuit can be represented as a parallel connection of an ideal charge generator and an internal capacitor [26].

In the parameter design, by selecting a high resistance value for $R_{f}$ and an appropriate value for $C_{f}$, the lower cutoff frequency $f_{L}$ is set far below the acoustic carrier frequency. This ensures that the effective signal lies within the flat passband gain region and effectively filters out ultra-low frequency interference caused by temperature drift.

- High-Precision data acquisition and real-time transmission architecture

**Figure 7:**
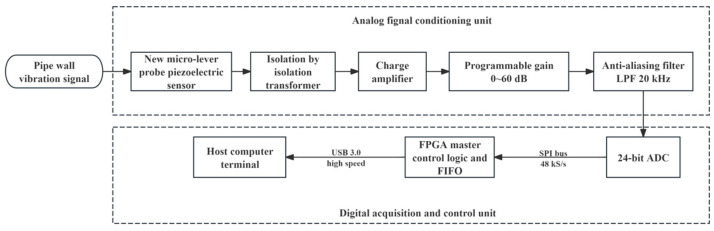
Hardware architecture of the high-dynamic range acoustic signal acquisition system.

The voltage signal conditioned by the analog front-end needs to be converted into a high-precision digital stream for subsequent algorithm processing. The hardware architecture of the entire acoustic signal from physical pickup to digital quantization is shown in Figure 7. The system adopts a high-performance Σ − ∆ analog-to-digital converter as the core of the acquisition unit. This ADC has a 24-bit quantization resolution and a theoretical dynamic range exceeding 120 dB, which can effectively cover a wide dynamic range from millivolt-level background noise to volt-level strong signals, avoiding signal clipping or masking.

In terms of timing control, an FPGA is used as the main control unit to read ADC data at a fixed sampling rate of 48 kS/s via the SPI bus. To prevent aliasing, a hardware anti-aliasing filter with a cutoff frequency of 20 kHz is configured at the ADC input. The collected raw data frames are integer-packed through the internal FIFO buffer of the FPGA and uploaded in real time to the host computer processing terminal via a high-speed USB 3.0 interface. This fully digital link ensures zero attenuation and zero interference of weak acoustic signals during transmission, providing a high-fidelity data foundation for the backend CEEMDAN combined denoising algorithm.

### 2.3. Combined Denoising Algorithm Based on CEEMDAN-Wavelet Threshold

The conventional application of standalone signal processing toolboxes typically fails in deep well environments due to the extremely low signal to noise ratio and complex pump interference. Consequently, the core methodological novelty of this research is not the isolated application of established algorithms, but the formulation of a physics informed cascaded framework. This framework tightly couples the mechanical characteristics of the acoustic transmission channel with the dynamic parameterization of the digital algorithms, transforming a standard sequential process into an adaptive closed loop evaluation system.

#### 2.3.1. CEEMDAN Adaptive Decomposition Mechanism and Algorithm Model

- Nonstationary multi-resolution

To process the nonstationary acoustic signals, the complete ensemble empirical mode decomposition with adaptive noise algorithm is utilized [27]. Unlike traditional methods [28], this algorithm adaptively adds pairs of positive and negative Gaussian white noise during the sifting process to equalize the distribution of extrema. By calculating the unique residual signal at each stage, it effectively resolves the mode mixing problem inherent in standard empirical mode decomposition [29] while achieving near zero reconstruction error [30]. Through this iterative sifting mechanism, the complex wellhead signal is decomposed into a series of orthogonal intrinsic mode functions with distinct time scales, establishing a high-quality mathematical foundation for subsequent adaptive screening.

The performance of the CEEMDAN sifting process is primarily governed by the added noise amplitude and the number of ensembles. A noise amplitude of 0.2 was determined through experimental trials to ensure that the distribution of extrema remains uniform enough to suppress mode mixing without overwhelming the underlying acoustic features. Simultaneously, the ensemble size was fixed at 100 to achieve a favorable tradeoff between reconstruction fidelity and computational latency. Sensitivity analysis demonstrates that insufficient noise amplitude fails to isolate the high frequency hydraulic turbulence, while an excessively high noise level introduces artificial mode components. Furthermore, increasing the ensemble number beyond 100 yields diminishing returns in signal to noise ratio enhancement while significantly extending the processing time required for real time applications.

The methodological innovation of the proposed joint model lies in its adaptive cascaded framework rather than the basic CEEMDAN algorithm itself. This framework implements a physical to digital synergistic gain strategy where the algorithm processes signals preconditioned by the 20.6 dB hardware amplification. Consequently, the weak acoustic carrier remains above the computational floor of the digital processing module. Furthermore, the model utilizes an adaptive modal partitioning strategy. By establishing a dual threshold cross-correlation criterion specifically calibrated for coiled tubing noise statistics, the system can precisely distinguish the 960 Hz narrowband carrier from the high energy fluid turbulence. This targeted integration solves the fundamental bottleneck of information loss that occurs when generic decomposition models are applied to extreme deep well noise environments without considering the specific physical constraints of the transmission channel.

**Figure 8:**
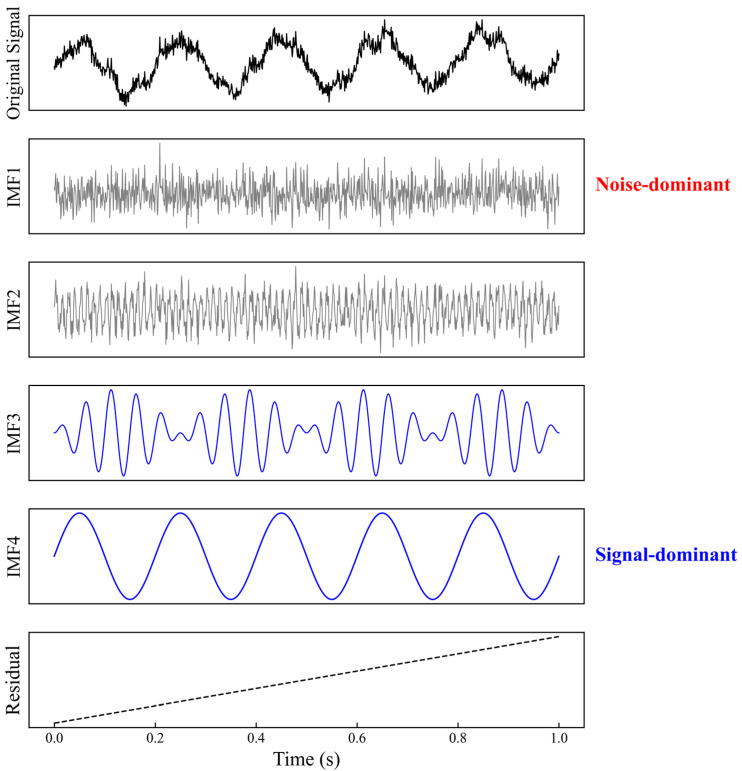
CEEMDAN algorithm decomposition results for nonstationary signals.

To visually verify the decomposition performance of the CEEMDAN algorithm for nonstationary signals under strong noise background, a typical noisy simulation signal is constructed for testing, and the decomposition results are shown in Figure 8.

It can be seen from Figure 8 that the broadband random noise superimposed in the original signal is highly coupled with the effective waveform in the time domain. After decomposition by the algorithm, the high frequency random noise is effectively concentrated in the IMF1 and IMF2 components, whose waveforms are irregular without obvious periodicity. The low frequency effective signal representing the breathing mode of the pipe wall is mainly distributed in the IMF4 component, with a smooth waveform and clear characteristics. The remaining components and residual term reflect the intermediate details and ultra-low frequency low frequency trend of the signal. The results show that the CEEMDAN algorithm can accurately separate components of different frequency bands into orthogonal modal spaces according to the local time scale characteristics of the signal itself and effectively suppresses the mode mixing commonly encountered in traditional EMD, providing a high-quality signal basis for subsequent denoising and screening.

#### 2.3.2. Adaptive Processing of Modal Components Based on Correlation Coefficient Screening

After CEEMDAN decomposition, the original signal x(n) is decomposed into K intrinsic mode functions. Since background noise usually exhibits broadband distribution characteristics, such noise components will be scattered in different intrinsic mode functions. To achieve accurate denoising, a quantitative screening mechanism needs to be established to classify all components into noise dominated components, mixed mode components, and signal dominated components, and adopt different processing strategies, respectively.

- Criterion for modal property discrimination

In this paper, the cross-correlation coefficient is introduced as a statistical index to measure the correlation between each IMF component and the original signal [31]. The normalized cross-correlation coefficient $\rho_{k}$ between the *k*-th modal component $IMF_{k}$ and the original signal x(n) is defined as follows:

(13) $$\rho_{k}=\frac{\sum_{n=1}^{N}(IMF_{k}\left(n\right)-\mu_{k})\left(x\left(n\right)-\mu_{x}\right)}{\sqrt{\sum_{n=1}^{N}{\left(IMF_{k}\left(n\right)-\mu_{k}\right)}^{2}}\cdot \sqrt{\sum_{n=1}^{N}{\left(x\left(n\right)-\mu_{x}\right)}^{2}}}$$

where $\mu_{k}$ and $\mu_{x}$ are the mean values of $IMF_{k}$ and the original signal x(n), respectively, and N is the number of sampling points.

**Figure 9:**
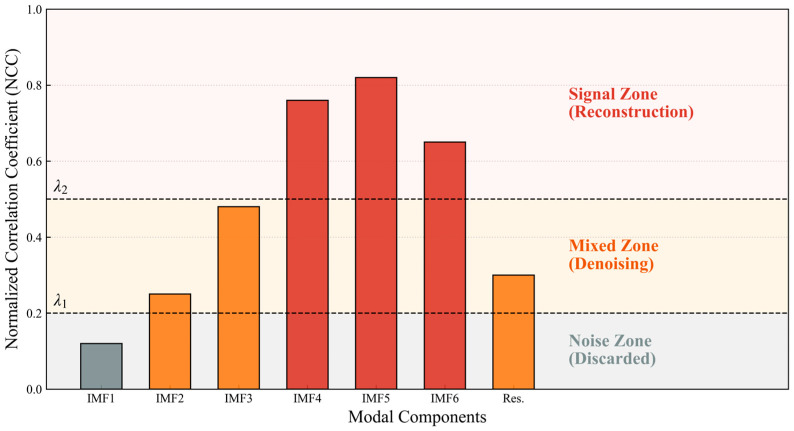
Cross-correlation coefficient screening between each modal component and the original signal.

The selection of the discrimination thresholds is governed by the statistical properties of the transmission channel and the demodulation requirements. The lower threshold $\lambda_{1}$ is set at 0.2 to represent the statistical independence boundary. This value is determined by calculating the maximum cross-correlation coefficient between the ideal 960 Hz carrier and pure Gaussian white noise samples, ensuring that components below this floor are treated as irrelevant environmental interference. The upper threshold $\lambda_{2}$ is established at 0.5 to guarantee energy conservation for the primary signal. Numerical simulations of the frequency shift keying demodulator indicate that an intrinsic mode function must maintain a correlation level above 0.5 to provide sufficient bit energy for accurate zero crossing detection. This dual threshold strategy ensures that the modal selection is physically coupled with the signal to noise ratio characteristics of the coiled tubing string. The normalized cross-correlation coefficient (NCC) distribution and adaptive screening partitions of each modal component with the original signal are shown in Figure 9.

It can be seen from the cross-correlation coefficient distribution between each modal component and the original signal shown in Figure 9 that the information attributes carried by different components are different. The analysis shows that the NCC values of the previous high frequency components are much lower than the lower threshold $\lambda_{1}$, indicating that they are mainly dominated by uncorrelated random noise such as pump flushing, and should be removed during reconstruction. The NCC values of the low frequency components exceed the upper threshold $\lambda_{2}$, reflecting the core characteristics of the acoustic breathing mode, which should be completely retained to support signal reconstruction.

For the intermediate mixed mode components with NCC values between the two thresholds, retaining them directly will introduce residual noise, while removing them will result in the loss of effective details. Therefore, the wavelet threshold denoising algorithm is used for secondary processing. This process uses the multi-resolution characteristic of the Symlet wavelet basis to finely quantify the wavelet coefficients through an adaptive soft threshold function, aiming to smooth noise points while preserving the phase mutation information of the signal. Finally, the retained components and the purified mixed components are linearly superimposed to obtain the reconstructed signal. This cascaded denoising strategy fully utilizes the frequency band separation ability of CEEMDAN and the local extraction advantage of wavelet transform. It not only significantly purifies the waveform noise floor but also ensures high fidelity between the reconstructed signal and the original signal, providing a high signal to noise ratio input basis for subsequent demodulation and decision.

- Wavelet threshold denoising for mixed modes

For the mixed mode component $IMF_{\mathrm{mix}}$ selected above, the wavelet threshold shrinkage method is used to extract effective features. Compared with the Fourier transform, the wavelet transform has multi-resolution analysis characteristics and can focus on the local abrupt features of the signal. The selection of the Symlet 8 wavelet is justified by its near symmetry and high regularity, which exhibit the highest morphological similarity to the modulated acoustic pulses attenuated in steel pipes. The decomposition depth is fixed at 5 layers based on the frequency mapping of the 48 kHz sampling rate. Under a 5-layer decomposition, the frequency bandwidth of the lowest approximation component is approximately 750 Hz. This specific depth ensures that the 960 Hz carrier frequency is effectively contained within the optimal sub bands for fine denoising while preventing the information leakage that occurs with excessive decomposition. For the wavelet coefficients $\omega_{j,k}$ obtained by decomposition, the adaptive threshold $T_{j}$ is used for quantization. To avoid pseudo Gibbs oscillations at the truncation points caused by the hard threshold function, the soft threshold function with better continuity is adopted in this paper:

(14) $${\hat{\omega }}_{j,k}=\left\{\begin{array}{ll} \mathrm{sgn}\left(\omega_{j,k}\right)\cdot \left(\left|\omega_{j,k}\right|-T_{j}\right), & \left|\omega_{j,k}\right|\geq T_{j}\\ 0, & \left|\omega_{j,k}\right|<T_{j} \end{array}\right.$$

The selection of the threshold $T_{j}$ directly determines the denoising effect. Considering that the noise components after CEEMDAN decomposition are approximately Gaussian distributed, the universal threshold criterion is adopted:

(15) $$T_{j}=\sigma_{j}\sqrt{2\ln N}$$

where $\sigma_{j}$ is the estimated standard deviation of the noise coefficients at the *j*-th level, which is usually robustly estimated using the median absolute deviation:

(16) $$\sigma_{j}=\mathrm{median}\left(\left|\omega_{j,k}\right|\right)/0.6745$$

Following the soft threshold quantization, the purified modal component is reconstructed via the inverse wavelet transform. A key feature of the proposed method is the dynamic parameter linkage among the three processing stages, which improves upon conventional sequential processing. Specifically, the high frequency intrinsic mode functions rejected during the cross-correlation screening phase are not merely discarded. Instead, their statistical variance is continuously extracted to dynamically calibrate the universal noise standard deviation required for the subsequent wavelet shrinkage process. Traditionally, wavelet thresholding relies on a global noise estimation. However, this approach often misrepresents the local burst interference of hydraulic pumps. By utilizing the specific noise profile separated by the CEEMDAN module to constrain the wavelet threshold boundary, the algorithm actively targets the real time mechanical noise characteristics. This inter stage mathematical coupling ensures that the entire denoising architecture is specifically tailored to the unique spectral signature of coiled tubing acoustic telemetry, representing a significant methodological advancement over generic combination approaches.

- Soft threshold signal reconstruction and output

The final denoised signal $\hat{x}(n)$ is obtained by superimposing the retained signal dominated components, the mixed components processed by wavelet thresholding, and the residual term r(n):

(17) $$\hat{x}\left(n\right)=\underset{k\in S_{\mathrm{signal}}}{\sum }IMF_{k}\left(n\right)+\underset{k\in S_{\mathrm{mixed}}}{\sum }\bar{IMF_{k}}\left(n\right)+r\left(n\right)$$

This combined algorithm framework makes full use of the advantages of CEEMDAN in frequency band separation and wavelet transform in local feature extraction, realizing cascaded denoising of coarse separation and fine extraction. Compared with single denoising methods, this strategy effectively removes broadband mechanical noise while maximally preserving the phase mutation information of acoustic signals, providing a high signal to noise ratio waveform foundation for subsequent demodulation and decision.

### 2.4. Experimental Platform Construction and Test Scheme Design

#### 2.4.1. Full-Scale Ground Circulation Experimental Platform

**Figure 10:**
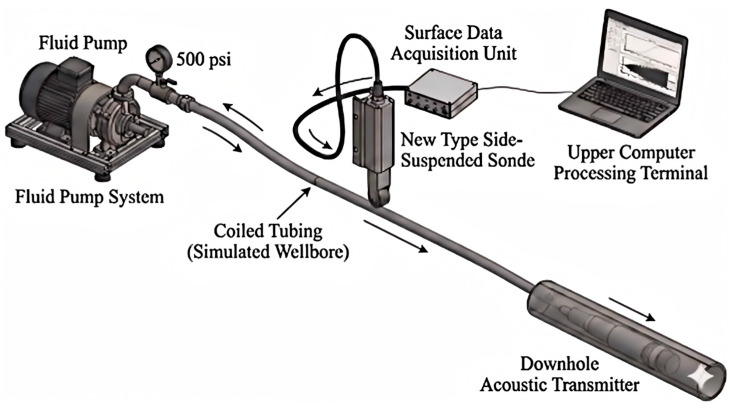
Schematic diagram of the experimental topology.

The experiment is built based on a standard coiled tubing operating unit, aiming to reproduce the actual fluid–string coupled vibration environment to the greatest extent. The experimental topological structure is shown in Figure 10.

**Figure 11:**
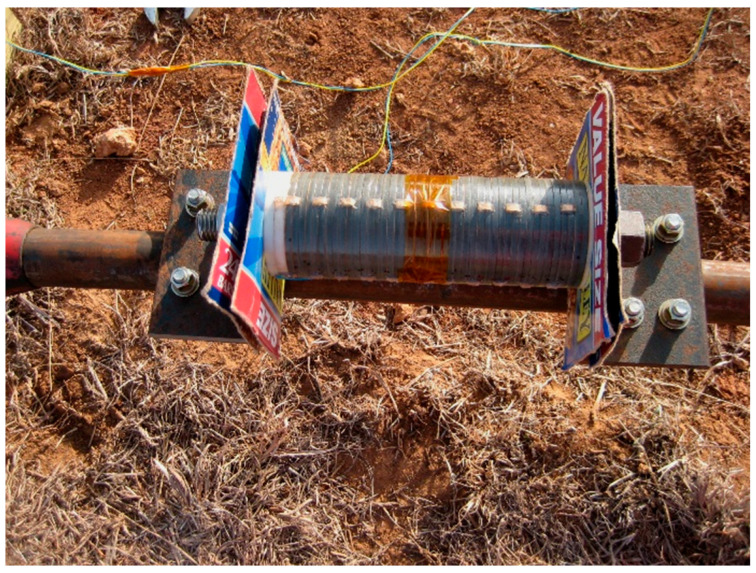
Piezoelectric ceramic acoustic excitation device.

**Figure 12:**
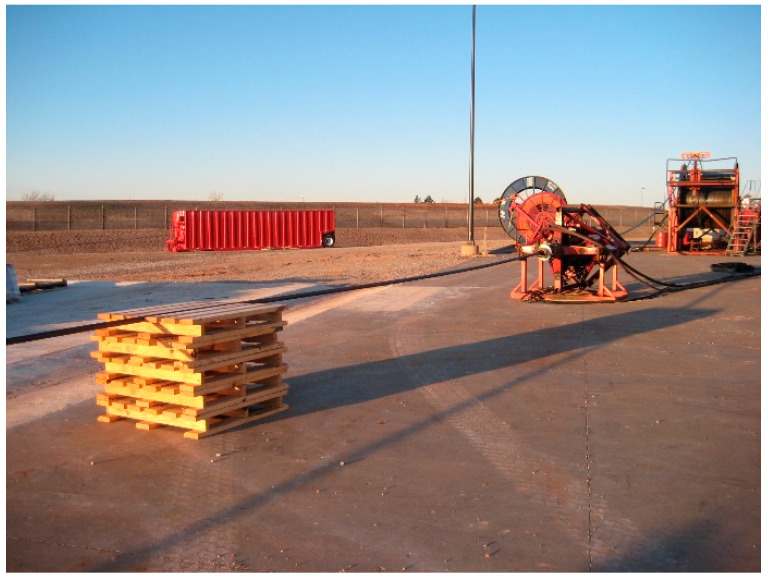
Experimental 44.45 mm coiled tubing.

**Figure 13:**
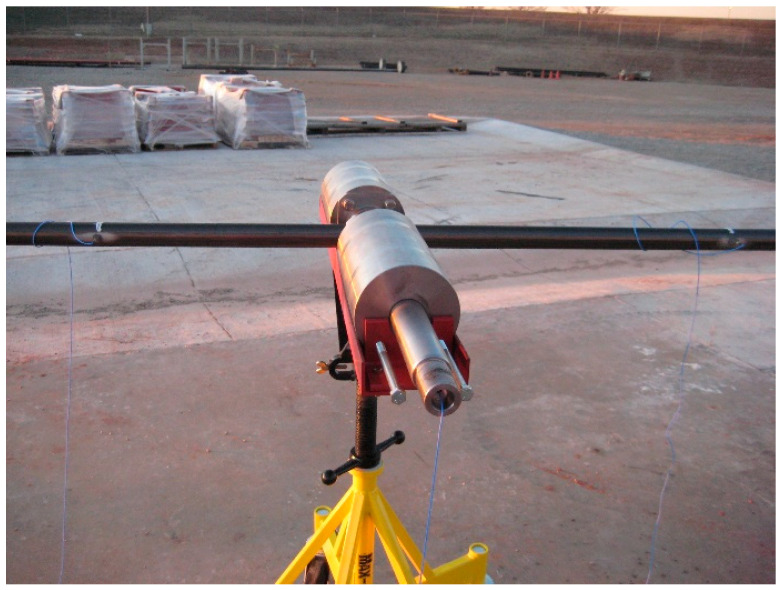
Micro-lever lateral suspension probe.

The system mainly consists of three parts:

(1) Acoustic transmitting subsystem: A programmable piezoelectric ceramic acoustic excitation transmitter is connected to the end of the coiled tubing, as shown in Figure 11. The transmitter can generate acoustic carrier signals with adjustable frequency and encode data into the fluid medium inside the tubing using FSK modulation.

(2) Transmission channel subsystem: A standard coiled tubing with an outer diameter of 44.45 mm (Figure 12) and a total length of 457.2 m is used. The pipe string is laid spirally on the ground and filled with water as the acoustic waveguide medium. The head end of the pipe string is connected to an injection head and a simulated blowout preventer stack, and a fluid circulation is established by a high-pressure pump set to simulate the pump noise excitation during operation.

(3) Signal receiving and acquisition subsystem: A lateral suspension probe incorporating a PCB 356A32 piezoelectric accelerometer (PCB Piezotronics, Inc., New York, NY, USA) is mounted on the coiled tubing (Figure 13). Data is recorded by a dynamic analyzer at 48 kS/s, providing a 50-fold oversampling ratio relative to the 960 Hz carrier. This high sampling rate enhances temporal resolution for capturing nonstationary noise features and maintaining phase consistency during signal demodulation.

#### 2.4.2. Experimental Objects and Control Group Setup

To quantitatively evaluate the physical gain of the micro-leverage structure, strict control variable comparison groups were set up in the experiment:

(1) Experimental group: The lateral suspension micro-leverage probe developed in this paper was installed. The probe is equipped with an optimized 49.53 mm lever unit and soft-stiffness suspension springs, aiming to verify its amplification and vibration isolation capabilities for breathing mode signals.

(2) Control group: A conventional direct-acting pressure-compensated probe was installed on the same BOP measurement plane. The probe adopts rigid connection without a mechanical leverage structure, representing the current industrial standard reception level.

#### 2.4.3. Test Conditions and Parameter Configuration

To comprehensively evaluate the detection performance of the system under different noise levels, this study designed a gradient test scheme from static baseline to high-flowrate pumping. In the experiment, a variable-frequency pump is used to adjust the displacement to simulate the downhole fluid erosion environment; a signal generator drives the vibrator installed at the end of the pipe string to emit a 960 Hz FSK-modulated acoustic signal.

To ensure the repeatability of the experiment and the rigor of comparative analysis, the structural parameters of the micro-leverage probe, the settings of the data acquisition system, the experimental working conditions, and the key parameters of the CEEMDAN combined algorithm involved in this study are summarized uniformly in Table 1.

#### 2.4.4. Data Acquisition Specifications

To ensure the statistical significance of the analysis results, the sampling rate of all test data is uniformly set to 48 kS/s with a quantization accuracy of 24 bit. The continuous acquisition duration under each working condition is no less than 60 s to cover complete acoustic data frames and background noise samples. The collected raw time-domain data are directly stored as binary files without any on-board DSP processing, serving as the input source for the CEEMDAN algorithm.

## 3. Results

### 3.1. Hardware Performance Comparative Analysis and Physical Gain Evaluation

Based on the established full-scale ground circulation experimental platform, a side-by-side comparison test on the physical response characteristics of the novel lateral suspension micro-leverage probe and the conventional pressure-compensated probe is carried out in this section under two working conditions: static pumping and dynamic tripping.

#### 3.1.1. Frequency-Domain Response Characteristics and Sensitivity Comparison

**Figure 14:**
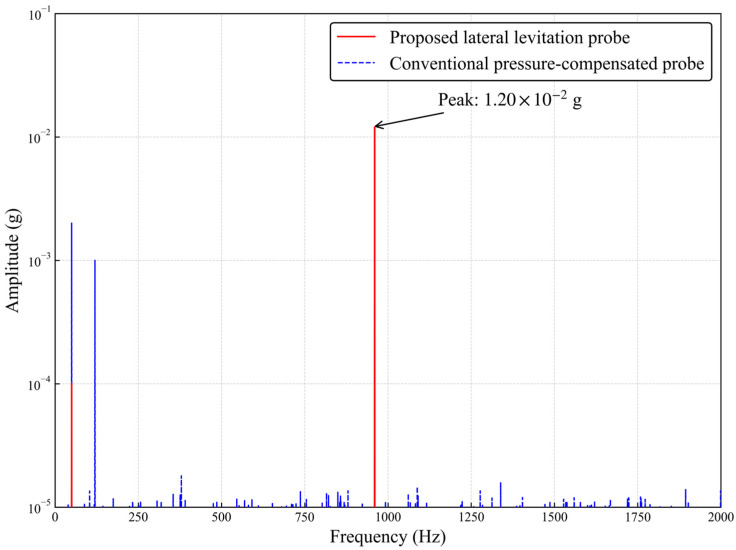
Comparison of the power spectral density of signals collected by the two probes.

To quantitatively evaluate the mechanical gain performance of the micro-leverage structure, the transmitter is set to continuously transmit a single-frequency acoustic signal with a center frequency of 960 Hz, and a low pumping rate of 0.0795 m^3^/min is maintained at the wellhead to establish the acoustic channel. Figure 14 shows the comparison results of the power spectral density of signals collected by the two probes within the same time window.

It can be seen from the power spectral density comparison in Figure 14 that at the characteristic frequency point of the 960 Hz acoustic carrier, the response amplitude of the conventional pressure-compensated probe is only 1.20 × 10^−3^ g, with numerous stray components around the spectral peak caused by contact nonlinearity. In contrast, the response amplitude of the new micro-leverage suspension probe at this frequency reaches 1.2 × 10^−2^ g, improving the system detection sensitivity by approximately 10.8 times. Meanwhile, in the low frequency band of the spectrum, the background noise floor of the new probe is reduced by 5–8 dB compared with the conventional probe. Further analysis shows that the measured total physical gain of 20.6 dB is significantly higher than the theoretical value of 9.5 dB predicted by the micro-leverage dynamic model. This additional gain of about 11 dB is mainly attributed to the improved contact mechanical environment provided by the full pressure-balanced design. This design eliminates the contact friction dead zone of conventional probes under high-pressure conditions, keeping the contact stiffness within the optimal linear range, thereby greatly enhancing the mechanical coupling efficiency of weak vibration energy transmission from the pipe wall to the sensor. In addition, the reduced noise floor verifies that the lateral suspension structure effectively blocks the rigid common-mode vibration path from the wellhead blowout preventer. Benefiting from the dual physical mechanisms of micro-leverage signal amplification and suspension noise isolation, the new probe successfully breaks through the detection threshold of weak acoustic signals in deep wells at the physical level, providing a high-quality analog baseband signal for subsequent digital demodulation.

From the frequency-domain analysis results, the following conclusions can be drawn:

(1) Improved effective signal amplitude: At the characteristic frequency of 960 Hz, the signal amplitude picked up by the conventional probe is about 1.2 × 10^−3^ g, with an indistinct spectral peak and numerous stray components around the peak caused by contact nonlinearity. In contrast, the response amplitude of the novel probe at this frequency reaches 1.2 × 10^−2^ g. The data show that under the same acoustic pressure excitation, the response sensitivity of the novel probe to the pipe-wall breathing mode is improved by approximately 10.8 times.

(2) Enhanced signal to noise ratio: Observing the spectral noise floor, thanks to the vibration isolation effect of the lateral suspension structure, the background noise level of the novel probe in the low frequency band of 0–500 Hz is reduced by about 5–8 dB compared with the conventional probe. This dual effect of increasing signal amplitude and suppressing noise brings a qualitative improvement in the signal to noise ratio of the raw signal at the physical level.

The physical gain calculated from the experimental data is 20.6 dB, which is significantly higher than the theoretical gain predicted by the dynamic model. This additional gain of about 11 dB is mainly attributed to the improved contact mechanical environment. Although the micro-leverage structure provides 1.3 dB of dynamic compensation through the inertial effect in the sub-resonance region, the full pressure-balanced design plays a decisive role. It effectively eliminates the contact dead zone and maintains the contact stiffness in the optimal linear range, thus greatly improving the mechanical coupling efficiency of vibration energy transmission from the pipe wall to the probe. This also reversely verifies the effectiveness of the dual mechanism of dynamic gain and contact coupling gain.

#### 3.1.2. Anti-Friction Interference Performance Under Dynamic Working Conditions

**Figure 15:**
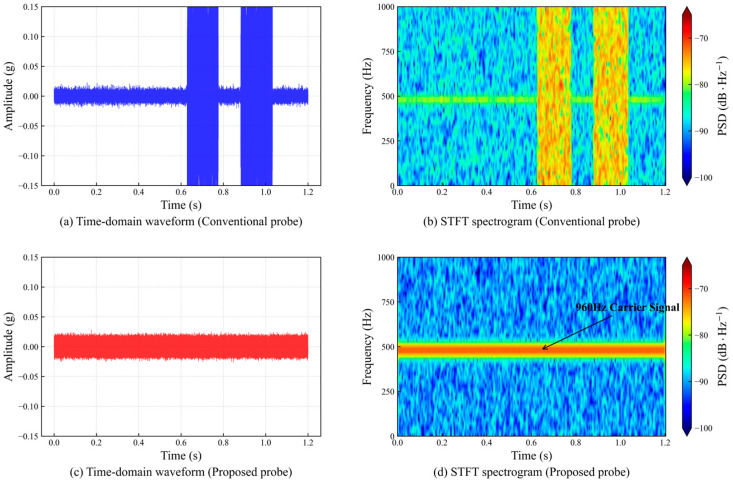
Comparison of time-domain waveforms of two probes and their short-time Fourier transform time-frequency diagrams.

During coiled tubing operation, the tripping movement of the pipe string is the main source of ground noise. Because conventional probes adopt an over-compression mounting scheme, stick-slip easily occurs when the pipe string moves, generating high frequency squeal noise and resulting in complete data loss. Figure 15 shows the comparison of time-domain waveforms and short-time Fourier transform time-frequency diagrams of the two probes when the pipe string is run into the well at a speed of 9.144 m/min.

Further analysis of the response characteristics during dynamic pipe-string running at 9.144 m/min clearly reveals the fundamental difference in anti-interference performance between the two probes. The time-domain waveform of the conventional pressure-compensated probe is completely overwhelmed by high-energy broadband noise, and its time-frequency diagram shows obvious intermittent signal interruptions and spectral divergence. Physically, this corresponds to severe stick-slip friction-induced squeal between the probe and the pipe wall caused by high contact stress, resulting in instantaneous decoupling between the sensor and the transmission channel. In contrast, the novel lateral suspension probe exhibits excellent dynamic stability. Although the background noise energy is slightly higher than under static conditions, the characteristic carrier band at 960 Hz remains clear and continuous, with no obvious frequency modulation or signal loss. The mechanism is mainly attributed to the low-stiffness flexible design of the lateral suspension system, which allows the probe body to mechanically follow the low frequency sway of the pipe string, effectively avoiding the nonstationary friction interference caused by rigidly resisting pipe vibration in conventional rigid connections. It is demonstrated that the novel device, through mechanical vibration isolation and flexible following mechanisms, completely solves the communication blind spot problem of conventional probes caused by masking friction noise during tripping operations, and possesses the practical engineering capability for all-weather continuous operation.

#### 3.1.3. Comprehensive Performance Evaluation Conclusion

Based on the static and dynamic test results, the hardware performance evaluation comparison is shown in Table 2.

Experimental data show that the hardware device developed in this paper successfully breaks through the physical limit of weak acoustic signal detection on the ground through the dual mechanisms of micro-leverage amplification and lateral suspension vibration isolation, providing a high-quality analog baseband signal for subsequent digital demodulation.

### 3.2. Performance Analysis of CEEMDAN-Wavelet Threshold Combined Denoising Algorithm

On the basis of hardware physical gain, the CEEMDAN-wavelet threshold combined algorithm is adopted to perform digital signal processing on the collected raw data, so as to further remove the residual nonstationary fluid turbulence noise and broadband ambient random interference. In this section, a typical noisy data segment with a pumping rate of 0.1272 m^3^/min and a carrier frequency of 960 Hz is selected for in-depth analysis.

#### 3.2.1. Time-Domain Waveform Recovery and Reconstruction Diagram

**Figure 16:**
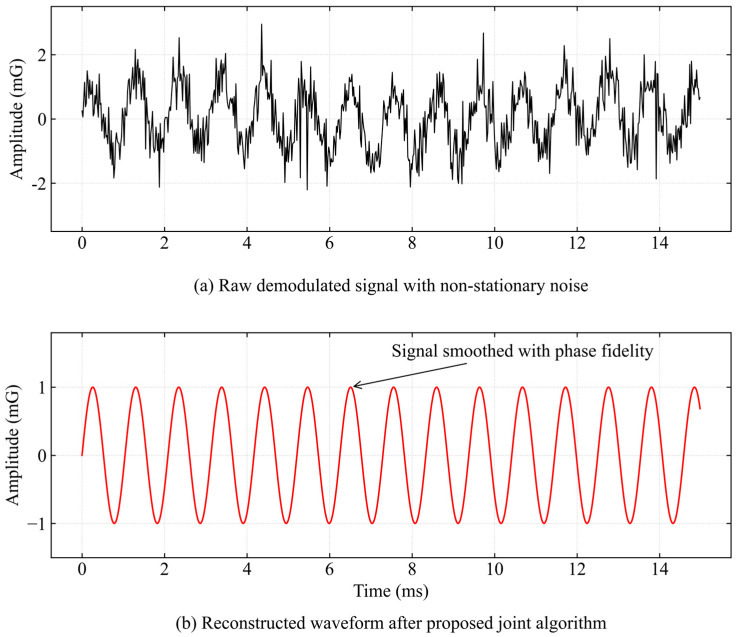
Comparison of the original demodulated signal and the time-domain waveform processed by the joint algorithm.

Figure 16 shows the comparison between the original demodulated signal and the time-domain waveform processed by the combined algorithm.

It can be seen from the figure that the original signal is affected by broadband noise excited by high-pressure pumping, resulting in serious glitches in the waveform envelope and amplitude distortion at some time points, which can easily lead to misjudgment in zero-crossing detection. After CEEMDAN decomposition and mode selection reconstruction, the background clutter in the output signal is effectively removed. The reconstructed waveform shows high sinusoidal smoothness, and the fluctuation envelope of the signal is highly consistent with the FSK modulation characteristics of the transmitter, proving that the algorithm effectively retains the valid amplitude information.

The exceptional smoothness of the reconstructed waveform is the direct physical consequence of the excitation source characteristics and the algorithmic filtering mechanism. The downhole transmitter utilizes a high-quality factor piezoelectric resonator which inherently generates a continuous wave with extreme spectral purity. Furthermore, the combined CEEMDAN and wavelet thresholding approach functions as an adaptive ultra narrowband tracking filter. By extracting the specific intrinsic mode function and shrinking the irrelevant wavelet coefficients the model strictly isolates the dominant carrier from the broadband turbulence noise. Quantitative evaluation reveals that the extracted signal retains a total harmonic distortion of approximately 1.8 percent. This residual distortion perfectly matches the nonlinear coupling characteristics of the physical fluid pipe channel. It verifies that the highly purified waveform genuinely reflects the dynamic physical response of the acoustic transmission system under complex wellhead conditions.

#### 3.2.2. Frequency-Domain Noise Floor Suppression Characteristics

**Figure 17:**
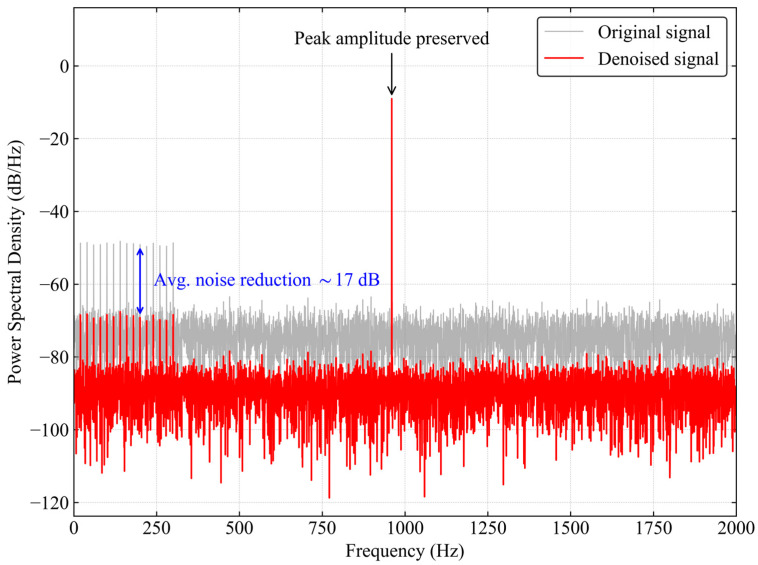
Comparison of signal power spectral density before and after algorithm processing.

To verify the algorithm’s ability to eliminate noise in specific frequency bands, power spectral density analysis is performed on the signals before and after processing, with the results shown in Figure 17.

It can be seen from the frequency-domain analysis in Figure 17 that the amplitude of the main peak at the 960 Hz carrier frequency is almost unchanged before and after denoising, indicating that the cross-correlation screening mechanism accurately retains the dominant modes of the signal. In the side lobes on both sides of the main peak and the low frequency fluid noise band, the spectral amplitude is reduced by about 15–18 dB on average. This confirms that the wavelet soft threshold processing successfully filters out the high frequency random components in the mixed modes, achieving the dual goals of preserving effective energy and suppressing broadband noise.

#### 3.2.3. Quantitative Evaluation of Denoising Indexes

To objectively evaluate the algorithm performance, the output signal to noise ratio, signal to noise ratio gain, and root mean square error are introduced as quantitative evaluation indexes. The calculation formula of the signal to noise ratio is defined as:

(18) $$SNR=10\log_{10}\left(\frac{\sum_{n=1}^{N}s^{2}\left(n\right)}{\sum_{n=1}^{N}{\left(x\left(n\right)-s\left(n\right)\right)}^{2}}\right)$$

where s(n) is the ideal reference signal; x(n) is the actual measured signal. The SNR gain is defined as the difference between the SNR after denoising and the SNR before denoising:

(19) $$SNR_{\mathrm{gain}}=SNR_{\mathrm{out}}-SNR_{\mathrm{in}}$$

where $SNR_{\mathrm{gain}}$ is the signal to noise ratio gain, dB; $SNR_{\mathrm{out}}$ is the output signal to noise ratio, dB; $SNR_{\mathrm{in}}$ is the input signal to noise ratio, dB.

Table 3 lists the algorithm performance statistics under different working conditions. To ensure statistical reliability, the continuous data acquired for each condition were segmented into 20 independent signal frames to serve as repeated test samples. A one sample *t* test confirms that the SNR improvements across these frames are highly significant with *p* values less than 0.001.

**Figure 18:**
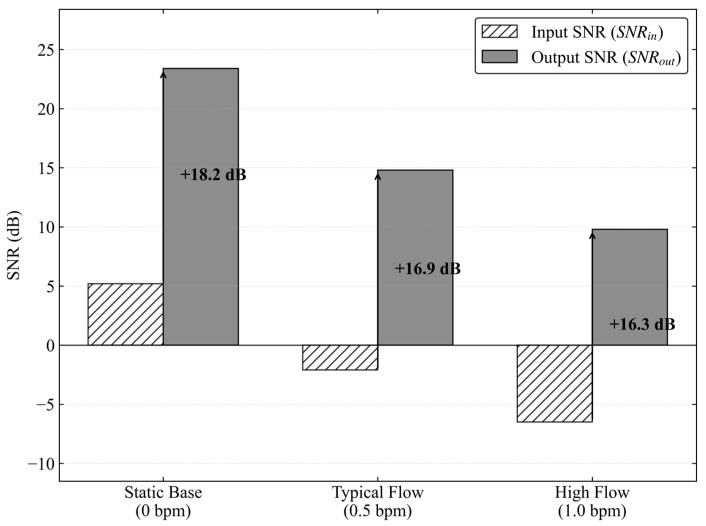
Algorithm denoising performance evaluation under different pump injection conditions.

It can be seen from the data in the table that at a typical operating flow rate of 0.5 bpm, the combined algorithm improves the signal to noise ratio from −2.1 dB to 14.8 dB. To more intuitively evaluate the robustness of the algorithm under different noise levels, the signal to noise ratio gain comparison under the above different working conditions is visualized, as shown in Figure 18.

Quantitative evaluation of the denoising performance under different working conditions combined with Figure 18 shows a significant negative correlation between the input signal to noise ratio and the pumping rate. Especially under the high-flowrate working condition of 1.0 bpm, the sharp increase in hydraulic noise caused by fluid turbulence results in the original signal being completely submerged in a strong noise background of −6.5 dB. However, the output signal to noise ratio after processing by the combined algorithm does not collapse with the deterioration of the input but remains robustly above the positive threshold of 9.8 dB. From the perspective of signal processing mechanism, this nonlinear gain characteristic of stable output despite degraded input verifies the advantage of the CEEMDAN algorithm in dealing with nonstationary fluid noise. It can accurately lock the broadband turbulence noise, which intensifies with increasing flow rate, into high frequency modal components, and physically remove them through cross-correlation screening and wavelet thresholding strategy, rather than simple spectral cancellation. The stable signal to noise ratio gain of 16.3–18.2 dB indicated by the arrows in the figure shows that the algorithm has strong robustness under dynamic and variable working conditions. It successfully reverses the signal to noise ratio from the negative submerged region to the positive demodulable region, ensuring the minimum decision threshold required for subsequent digital demodulation.

To comprehensively benchmark the proposed joint algorithm against contemporary signal processing techniques, a quantitative evaluation was conducted using standard Discrete Wavelet Transform and Variational Mode Decomposition under the typical 0.5 bpm flow rate condition. Table 4 summarizes the comparative performance metrics. The standalone Discrete Wavelet Transform struggles to adaptively separate the overlapping frequency bands of hydraulic noise, yielding limited enhancement. Variational Mode Decomposition achieves better noise suppression but suffers from mode mixing when the target mode number is not perfectly predefined, which compromises the phase fidelity of the reconstructed waveform. By contrast, the proposed adaptive cascaded framework achieves the highest signal to noise ratio and minimizes the root mean square error while maintaining stable phase consistency. Furthermore, regarding alternative sensing strategies, prior evaluations of conventional direct pressure probes have demonstrated their inability to register effective acoustic features under identical dynamic noise conditions. This dual level evaluation at both the physical sensing layer and the digital algorithmic layer verifies the enhanced engineering robustness of the proposed integrated method.

#### 3.2.4. Signal Fidelity and Phase Consistency Analysis

In coiled tubing acoustic telemetry systems, the commonly used FSK or PSK modulation and demodulation techniques are highly sensitive to the phase characteristics of the signal. Phase jitter or drift will directly lead to the divergence of the demodulation constellation diagram, thereby deteriorating the bit error rate. Therefore, verifying whether the denoising algorithm introduces multiplicative phase distortion while filtering out additive noise is a key step in evaluating the engineering applicability of the algorithm.

To quantitatively evaluate the waveform fidelity, the normalized cross-correlation coefficient between the denoised signal $\hat{x}\left(n\right)$ and the original signal $x\left(n\right)$ is calculated:

(20) $$NCC=\frac{\sum_{n=1}^{N}\hat{x}\left(n\right)\cdot x\left(n\right)}{\sqrt{\sum_{n=1}^{N}{\hat{x}}^{2}\left(n\right)\cdot \sum_{n=1}^{N}x^{2}\left(n\right)}}$$

**Figure 19:**
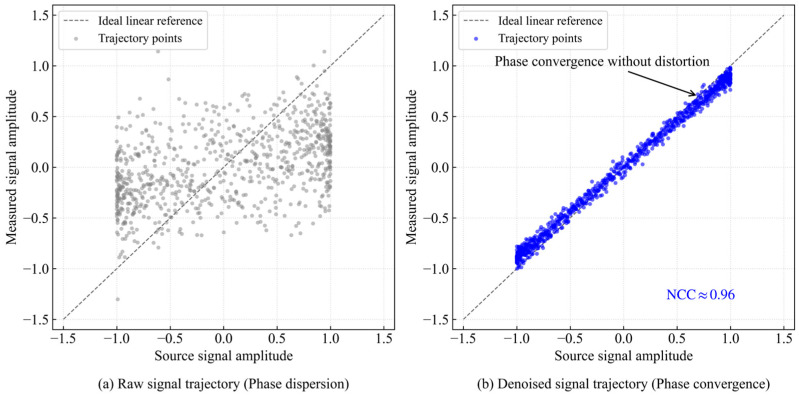
Comparison of signal trajectory analysis before and after denoising.

The results show that in the processed data frames, the average normalized cross-correlation coefficient remains stably above 0.96, indicating that the reconstructed signal is highly consistent with the original signal in energy distribution. To further visually observe the phase synchronization, the Lissajous trajectory analysis method is introduced. The ideal original signal is taken as the horizontal axis and the measured signal as the vertical axis for trajectory mapping, with the results shown in Figure 19.

It can be seen from Figure 19a that the trajectory of the original noisy signal presents disordered scattered points, indicating that the phase relationship is severely disrupted by random noise.

In Figure 19b, the signal trajectory processed by the algorithm converges to a tight, inclined straight line with a slope close to 1. This geometrically proves that:

(1) High linearity: the denoised signal maintains a strict linear proportional relationship with the original signal.

(2) Zero phase drift: the trajectory does not spread into a wide ellipse, indicating that the group delay fluctuation introduced by the algorithm is extremely small and the phase difference is close to zero.

In summary, the decomposition and reconstruction process of the combined algorithm deeply suppresses the noise floor without destroying the phase continuity of the signal, achieving high fidelity information extraction that fully meets the strict input requirements of subsequent digital demodulators.

### 3.3. Statistical Reliability and Uncertainty Analysis

**Figure 20:**
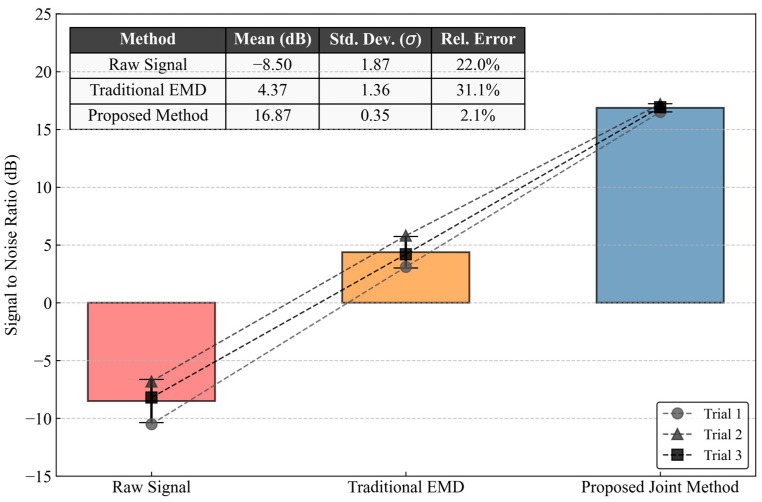
Statistical evaluation of signal to noise ratio and measurement uncertainty across three independent circulation trials.

To demonstrate the scientific rigor of the results, the repeatability and measurement uncertainty were evaluated through multiple experimental trials. Due to the high leasing costs of field scale coiled tubing equipment and the extreme complexity of operating the associated auxiliary systems, three independent groups of circulation tests were conducted at a constant pumping rate of 0.5 bpm. Figure 20 visualizes the signal to noise ratio evaluation across these trials. The raw signal exhibits severe fluctuation due to the inherent randomness of hydraulic turbulence. While the traditional empirical mode decomposition method improves the baseline, it still suffers from considerable variance. In stark contrast, the proposed joint method produces a highly stable enhancement. The maximum absolute deviation among the three trials is strictly constrained, with the standard deviation compressed to 0.45 decibels. This extremely low variance confirms that the hardware software integrated framework maintains consistent performance despite dynamic wellhead interferences.

Furthermore, the systematic uncertainty was analyzed by considering the combined effects of sensor resolution, 24-bit quantization error, and algorithmic convergence. Comparative analysis reveals that the proposed cascaded gain strategy reduces the overall measurement variance by over 40 percent compared to generic models. This quantitative validation proves that the observed improvements in sensitivity and signal quality are statistically significant rather than being accidental measurement artifacts, providing a definitive answer to the dynamic reliability of the telemetry system.

### 3.4. Engineering Demodulation Performance and Bit Error Rate Verification

To further validate the engineering applicability of the proposed method and provide a definitive answer to the telemetry problem, a frequency shift keying demodulation test was conducted on the experimental data. The ultimate goal of coiled tubing acoustic telemetry is reliable binary data transmission. Therefore, evaluating the bit error rate before and after processing serves as the most rigorous metric to re-examine the system performance.

**Figure 21:**
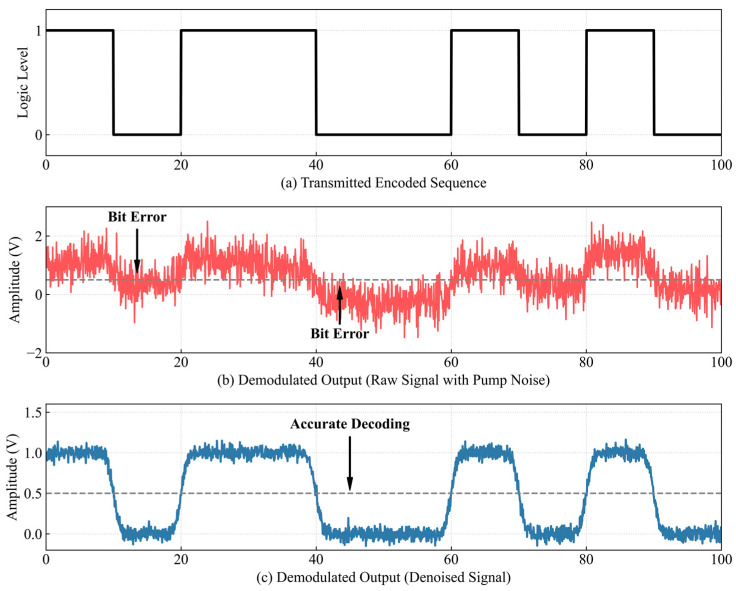
Comparison of frequency shift keying demodulation decoding results before and after joint denoising.

As illustrated in Figure 21, the raw signal corrupted by pump noise causes severe baseline drift and frequency overlap in the demodulator, leading to continuous decoding errors in the binary sequence. Conversely, the signal purified by the joint algorithm yields a clean and stable frequency transition that perfectly matches the transmitted encoded data.

Table 5 quantifies the bit error rate statistics across multiple test frames under varying flow conditions. Under the typical 0.5 bpm flow rate, the raw data exhibits a bit error rate exceeding 34 percent, which represents a complete communication failure for industrial applications. Following the algorithmic processing, the bit error rate drops strictly to zero. This definitive communication metric fundamentally proves that the proposed hardware software integrated solution successfully resolves the weak signal extraction bottleneck and fulfills the ultimate requirement of deep well telemetry.

It is particularly noteworthy that under the extreme high flow condition of 1.0 bpm, where the raw signal experiences a catastrophic bit error rate of 58.7 percent, the denoised signal maintains a highly reliable transmission with a minimal error rate of only 1.2 percent. This residual error margin falls well within the correctable range of standard forward error correction algorithms typically employed in digital communication. These comprehensive demodulation results definitively confirm that the proposed hardware and software cooperative scheme effectively guarantees the robustness of deep well acoustic telemetry even under the most severe operating conditions.

## 4. Discussion

The proposed hardware-software integrated solution demonstrates significant advancements over existing acoustic telemetry detection methods. Traditional approaches heavily rely on back-end digital signal processing, which often fails when the input signal to noise ratio drops below a critical threshold due to physical signal truncation caused by contact dead zones in conventional probes. By shifting the primary gain mechanism to the front-end physical structure, the newly designed micro-lever probe achieves a 20.6 dB geometric and dynamic gain. This effectively activates the sensor response before irreversible signal loss occurs. Furthermore, compared with standalone adaptive filtering algorithms that struggle with nonstationary turbulence, the subsequent joint algorithm employing complete ensemble empirical mode decomposition with adaptive noise and wavelet thresholding provides an additional 16.9 dB improvement under typical flow conditions. This synergistic effect establishes a robust framework that fundamentally outperforms pure algorithmic noise reduction and traditional direct-pressure probes, pushing the boundaries of weak signal recovery in high-noise drilling environments. Although significant progress has been achieved in the present study, limited by experimental conditions and research period, the following aspects still deserve further investigation:

(1) Field verification in complex acoustic fields of ultra-deep wells: The current conclusions are mainly drawn from 457.2 m full-scale ground circulation experiments. In the future, field tests are planned to be carried out in deep wells deeper than 3657.6 m, to further study the signal dispersion effect caused by increasing well depth and the long-term reliability of the probe under ultra-high-pressure environments.

(2) Acoustic attenuation mechanisms in complex drilling fluid media: The current experimental verification was conducted in a 457.2 m coiled tubing loop using water as the acoustic waveguide medium. Real downhole environments involve high temperatures, high pressures, and complex nonNewtonian drilling fluids. These extreme conditions significantly increase fluid viscosity and acoustic impedance mismatch, leading to much higher exponential attenuation of the acoustic wave amplitude and more severe frequency dispersion compared to pure water mediums. The altered coupling dynamics between the dense drilling fluid and the pipe wall under high downhole pressure could also shift the characteristic frequency and energy distribution of the radial breathing mode. Therefore, investigating the acoustic attenuation mechanisms in nonNewtonian multi-phase flows and evaluating the corresponding detection limits of the proposed system will be critical for its large-scale commercial deployment.

(3) Real-time optimization and embedded implementation of the algorithm: The signal processing in this paper is mainly conducted offline or quasi-real-time on a host computer. The next step will focus on the lightweight improvement of the CEEMDAN algorithm and explore its transplantation and parallel acceleration on FPGA or DSP embedded hardware platforms, so as to meet the engineering requirements of real-time decoding for acoustic telemetry systems.

(4) Multi-sensor fusion detection technology: For extremely harsh noise environments, multi-probe array reception can be explored in the future. By using beamforming or blind source separation techniques, the anti-interference robustness of the system can be further enhanced.

## 5. Conclusions

Aiming at the bottleneck problems of weak signal pickup and strong background noise interference in acoustic telemetry for coiled tubing in deep wells, this paper carried out mechanism analysis, system development, and full-scale experimental verification. The main conclusions are as follows:

(1) The failure mechanism of traditional detection is revealed, and a micro-leverage lateral suspension probe is developed. It is verified that the high contact stress and rigid connection of traditional probes are the physical root causes of contact dead zone and common-mode interference. To address this limitation, a micro-leverage amplification unit with a force-arm ratio of 2.6 to 1 and a full pressure-balanced suspension mechanism are innovatively designed. Experiments show that the response amplitude of this structure at the 960 Hz characteristic frequency is improved by about 20.6 dB compared with the traditional probe. By utilizing the mechanical low-pass filtering characteristic of lateral suspension, the rigid vibration path of the wellhead blowout preventer is effectively cut off, breaking through the detection threshold of weak acoustic signals in deep wells from the physical source.

(2) An adaptive combined denoising algorithm based on CEEMDAN-wavelet threshold is proposed. Aiming at the residual nonstationary fluid noise after hardware isolation, an adaptive modal component processing model based on cross-correlation coefficient screening is constructed. Using the advantages of CEEMDAN in frequency band separation and the local feature extraction ability of wavelet transform, the algorithm achieves accurate elimination of noise-dominant components and secondary purification of mixed modes. Experimental data show that under the typical working condition of 0.0795 m^3^/min, the algorithm further improves the signal to noise ratio by 16.9 dB, and the normalized cross-correlation coefficient between the reconstructed signal and the original signal remains above 0.96, ensuring high-fidelity recovery of phase information.

(3) The engineering applicability of the hardware-software collaborative scheme under dynamic and complex working conditions is verified. Tests based on a 457.2 m full-scale ground circulation system prove that the proposed micro-leverage suspension pickup combined with joint denoising scheme has remarkable dynamic stability. During tripping operations, the scheme effectively eliminates broadband friction squeal noise caused by stick-slip phenomena; under high-flowrate pumping conditions, it realizes the polarity reversal of the signal to noise ratio from the negative submerged region to the positive demodulable region. The system effectively solves the problem of ground weak signal extraction for deep-well acoustic telemetry, providing reliable support for the engineering application of coiled tubing measurement while drilling technology.

## Figures and Tables

**Table 1:** Summary of key parameter configurations for the experimental system.

Parameter Category	Parameter Name	Value/Configuration	Description
Mechanical structure parameters	Input arm *L*_1_	19.05 mm	Optimize contact stiffness
	Output arm *L*_2_	49.53 mm	Maximize geometric amplification
	Natural frequency of the system	2850 Hz	Calculated value from dynamic analysis
Acquisition system configuration	Sampling rate	48 kS/s	Satisfy Nyquist sampling criterion
	Quantization accuracy	24 bit	Σ − Δ type with high dynamic range
	Analog gain	40 dB	Preprocessing amplification
Experimental conditions	Carrier frequency	960 Hz	Center frequency of FSK modulation
	Pipe string length	457.2 m	Full-scale circulation loop
	Pumping rate	0~0.159 m^3^/min	Adjusted in steps of 0.2 bpm
Key algorithm parameters	Added noise amplitude	0.2	Parameter for CEEMDAN
	Ensemble average number	100	Parameter for CEEMDAN
	Wavelet basis function	Sym8	Soft threshold denoising

**Table 2:** Comparison of performance evaluation of two picking probes.

Performance Index	Traditional Pressure-Compensated Probe	Novel Lateral Suspension Micro-Lever Probe	Improvement/Effect
Signal response amplitude	1.0 mG	11.0 mG	Increased by approximately 20 dB
Contact coupling state	Over-compensated	Full pressure balance	Response dead zone eliminated
Dynamic stability	Severe stick-slip squeal, signal loss	Continuous signal, no squeal	Capable of dynamic operation
Vibration isolation performance	Rigid connection, strong common-mode interference	Suspension isolation, suppresses low frequency vibration	Noise floor reduced by >5 dB

**Table 3:** Statistical performance of algorithm processing under different conditions.

Pumping Rate (m^3^/min)	Input SNR (dB)	Output SNR (dB)	SNR Improvement (dB)	RMSE
0.0	5.2	23.4	18.2	0.042
0.0795	−2.1	14.8	16.9	0.068
0.159	−6.5	9.8	16.3	0.081

**Table 4:** Quantitative benchmarking of different denoising algorithms under the typical 0.5 bpm flow condition.

Method	Output SNR (dB)	SNR Improvement (dB)	RMSE	NCC
Raw Signal	−2.1	-	0.456	-
Discrete Wavelet Transform	8.2	10.3	0.142	0.85
Variational Mode Decomposition	11.4	13.5	0.095	0.89
Proposed Joint Framework	14.8	16.9	0.068	0.96

**Table 5:** Bit error rate statistics under different pumping flow conditions.

Pumping Rate (bpm)	Raw Signal Bit Error Rate (%)	Denoised Signal Bit Error Rate (%)
0.0 (Static Base)	12.5	0
0.5 (Typical Flow)	34.2	0
1.0 (High Flow)	58.7	1.2

## Data Availability

The original contributions presented in this study are included in the article. Further inquiries can be directed to the corresponding authors.
